# *ETV6-RUNX1* Rearrangement in Tunisian Pediatric B-Lineage Acute Lymphoblastic Leukemia

**DOI:** 10.1155/2009/924301

**Published:** 2009-12-22

**Authors:** Abir Gmidène, Hatem Elghezal, Hlima Sennana, Yosra Ben Youssef, Balkiss Meddeb, Moez Elloumi, Abderrahim Khlif, Ali Saad

**Affiliations:** ^1^Laboratoire de Cytogénétique et de Biologie de la Reproduction, CHU Farhat Hached, Sousse 4000, Tunisia; ^2^Service d'Hématologie, CHU Farhat Hached, Sousse 4000, Tunisia; ^3^Service d'Hématologie, CHU Aziza Othmana, Tunis 1008, Tunisia; ^4^Service d'Hématologie, CHU Hédi Chaker, Sfax 3029, Tunisia

## Abstract

In this study, Forty-one out of fifty-seven Tunisian children with B-lineage acute lymphoblastic leukemia (B-ALL), and without cytogenetically detectable recurrent abnormalities at the time of the diagnosis, were evaluated by fluorescence in situ hybridization (FISH) for the t(12;21). This translocation leads *ETV6-RUNX1* (previously *TEL-AML1*) fusion gene. 16 patients (28%) had *ETV6-RUNX1* rearrangement. In addition to this rearrangement, two cases showed a loss of the normal *ETV6* allele, and three others showed an extra signal of the *RUNX1* gene. Seven patients without *ETV6-RUNX1* rearrangement showed extra signals of the *RUNX1* gene. One out of the 7 patients was also associated with a t(3;12) identified by FISH. This is the first Tunisian study in which we report the incidence of t(12;21) among childhood B-lineage ALL and in which we have found multiple copies of *RUNX1*. Finally, our findings confirm that additional or secondary genetic changes are commonly encountered in pediatric B-lineage ALL with *ETV6-RUNX1* gene fusion which is envisaged to play a pivotal role in disease progression.

## 1. Introduction

The t(12;21)(p13;q22) translocation is the most common genetic alteration in childhood B-cell acute lymphoblastic leukemia occurring in approximately 25% of cases and is associated with a favorable outcome [1, 2]. 

The molecular consequence of this translocation is the fusion of two known genes:* ETV6* mapped to 12p13 and *RUNX1* located at 21q22 [1, 3]. It results in the formation of a hybrid protein involving the helix-loop-helix domain of *ETV6* and the entire *RUNX1* gene that appears to interfere with transactivation by the normal *RUNX1* in a transdominant manner [3, 4].

Because it is a cryptic translocation which usually escapes diagnosis on conventional cytogenetic (CC) study due to the similarity of the changed bands, molecular cytogenetic tools such as fluorescence in situ hybridization (FISH) are needed to determine the incidence of the *ETV6-RUNX1* fusion in the Tunisian B-lineage ALL pediatric cases that had normal karyotypes or random chromosome aberrations at diagnosis and to compare our findings to those previously described in literature.

## 2. Methods

### 2.1. Patients

Among 57 childhood B-lineage ALL cases analyzed by conventional karyotype, 41 were selected for FISH analysis according the following criteria: age (0–16); absence of cytogenetically detectable recurrent abnormalities, that is, high hyperdiploid karyotypes (51–65 chromosomes), t(4;11), t(1;19), and t(9;22). Theses patients are referred to our laboratory for cytogenetic analyses from different hematological services in Tunisia.

### 2.2. Conventional Cytogenetics

Chromosomal preparations with RHG-banding were performed according to a previously described protocol [5].

Chromosomes were identified and arranged according to the international system for human cytogenetic nomenclature (ISCN 2005) [6], and the karyotype profile was determined by the analysis of at least 20 metaphases.

### 2.3. Fluorescence In Situ Hybridization (FISH)

The presence of the *ETV6-RUNX1* fusion gene was assessed using two YAC clones (the YAC936E2 and the YAC821S11) selected from their location in the 12p13 (*ETV6*) and the 21q22 (*RUNX1*) regions, respectively, in the “Centre d'Etude du Polymorphisme Humain Database” (http://www.cephb.fr/). 

The *ETV6* and the *RUNX1* probes were labeled with tetramethylrhodamine-6-dUTP and fluorescein 12-dUTP (Abbott-Vysis, Downers Grove, IL, USA), respectively, using nick translation kit (Abbott-Vysis, Downers Grove, IL, USA).

For FISH technique, slides were denatured in 70% formamide at 75°C for 1 minute and 30 seconds then dehydrated with cold (−20°C) 70%, 85%, and 100% ethanol for 1 minute and 30 seconds each.

After drying, the *ETV6-RUNX1* dual-color FISH probe, denatured as preliminary at 75°C for 5–10 minutes, was applied into a slide, covered with a 24 × 24 mm coverslip and sealed with a rubber cement. Slides were then placed in a humid light-proof container at 37°C for overnight hybridization. 

After hybridization, the coverslip was removed and slides were washed in 0.4 standard saline citrate (SSC) for 5 minutes at 75°C, followed by a second wash in 2XSSC-0.1% NP40 for 2 minutes.

 After drying, the slides were counterstained with 4′,6-diamidino-2-phenylindole and examined with a fluorescent microscope equipped with appropriate filters and CytoVision FISH system image capture software (Zeiss Axioskop 2 plus) and at least 50 metaphase cells, and 100 interphase nuclei were analyzed for *ETV6-RUNX1* translocation.

**Figure 1:**
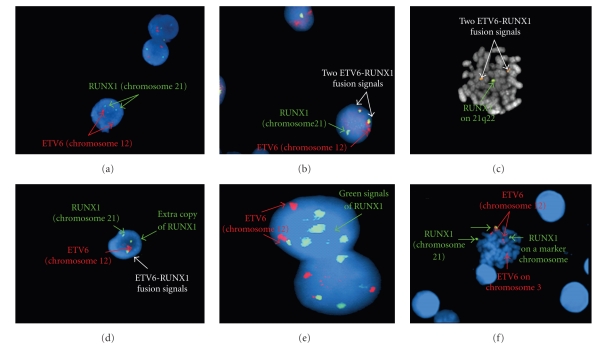
(a) an interphase cell with no translocation, (b) An interphase cell with two *ETV6-RUNX1* fusion signals (yellow), (c) a metaphase cell with an *ETV6-RUNX1* fusion signal and deletion of the second *ETV6* allele (missing a red signal from the cell), (d) an interphase cell with *ETV6-RUNX1* fusion signal and an extra copy of the *RUNX1* gene, (e) two interphase cells with extra copies of the *RUNX1* gene and without *ETV6-RUNX1* fusion, (f) t(3;12) with a marker chromosome that harbored an extra copy of the *RUNX1* gene.

 In a normal case, the hybridization with the *ETV6* (red) and the* RUNX1* (green) probes showed two red and two green signal patterns (Figure 1(a)). However, in a case with the t(12;21), the hybridization with these probes showed one or two fusion signals (red/green or yellow), one red and one green signal patterns corresponding to the normal copies of the *ETV6 *and the *RUNX1* genes, respectively, (Figure 1).

## 3. Results

Among 57 children with B-lineage ALL, 16 cases were not screened for this study (4 cases with t(9;22)(q34;q22) from which one was also associated to a hyperdiploid karyotype with 53 chromosomes, 1 case with t(1;19)(q23;p13), 1 case with t(8;14)(q24;q32), 2 cases with t(4;11)(q21;q23), 2 cases with del(9)(p12p23), 1 case with del(11)(q23), 3 cases with del(6q), and 2 cases with high hyperdiploidy (53 and 56 chromosomes). Both are associated to an add(14)(q32).

The selected 41 cases included 25 males and 16 females with age ranging from 1.2 to 15 years (mean 7.4 years). 

 Twenty-three out of the 41 patients (56%) had abnormal FISH findings (Table 1).

FISH analysis disclosed 16 positive cases for *ETV6-RUNX1* fusion gene among 57 pediatric B-lineage ALL cases (28%). 13 presented a normal karyotype (cases 1, 2, 4, 9, 10, 11, 12, 13, 14, 15, 19, 21, 22) and 2 had random chromosomal aberrations (cases 3 and 18), and in the remaining case, it was not possible to obtain adequate chromosomal preparations (case 20). Two of those sixteen patients (cases 1 and 3) had two fusion signals (Figure 1(b)).

Additional chromosomal aberrations accompanying the t(12;21) were found in five patients (31.2%). Deletion of nontranslocated *ETV6* allele was detected in two patients (cases 1 and 2) (Figure 1(c)), and three patients without +21 showed extra signal of *RUNX1* (cases 4, 14, and 21) (Figure 1(d)). Those include a case in which the clone with the extra signal of *RUNX1* was distinct from the one harboring *ETV6-RUNX1* fusion gene (case 21, Table 1).

Extra copies of* RUNX1* gene were also detected in 7 cases without t(12;21); three of which (cases 8, 17, and 23) do not have +21 on the karyotype. In the four cases remaining (cases 5, 6, 7, and 16) the number of *RUNX1* signals detected by FISH was in agreement with the number of chromosome 21 or the presence of marker chromosomes (Figure 1(e)). Of the cases, one (case 5) was associated with t(3;12) detected by metaphase FISH (Figure 1(f)).

## 4. Discussion

In the present study, we used FISH for the detection of *ETV6-RUNX1* fusion gene in 41 children with B-lineage ALL that had normal karyotypes or random chromosome aberrations at diagnosis. To our knowledge, this is the first report of the frequency of the t(12;21) from Tunisia and the second from an Arab country after the Egyptian study published by Mikhail et al. in 2002 [7].

*ETV6-RUNX1* rearrangement was present in 16 of the 57 selected subgroup of Tunisian patients with B-lineage ALL (28%). Of them, 13 (13/16, 81%) had a normal karyotype at diagnosis, a percentage higher than previously reported by Veiga et al. in Brazil (6/12, 50%) [8] and by Douet-Guilbert et al. [9] in France (2/10, 20%).

We confirm so the efficiency of FISH technique in the detection of such cryptic chromosomal rearrangement, usually missed by CC [10, 11].

In Egypt, UK, India, Korea, and Malaysia,* ETV6-RUNX1* rearrangement was found with a lower frequency than we have found, ranging from 0 to 22% [7, 12–14]. 

However, in B-lineage ALL patients of Germany [11], Italy [11], and France [15], the frequency of this translocation is reported to be about 25%. A frequency of 31% is found among American patients [16], and we recently report in Israel [17] and in Brazil [8] an incidence of 40%. 

This difference could be explained by the fact that due to the expected peak incidence at 3–5 years reported in the majority of the published series, our cases could have been biased for old age (mean age 7.4 years), which then corresponds to lower t(12;21) incidence.

The *ETV6-RUNX1* fusion gene on the der(21) was detected in all patients with t(12;21), whereas the reciprocal *ETV6-RUNX1* fusion gene on the der(12) was observed in only two cases. 

While this t(12;21) may initiate the leukemic process, critical secondary genetic events found in association with *ETV6-RUNX1* fusion gene are currently believed to be pivotal for leukemogenesis and are previously reported in literature [4, 18–20].

In our study, additional chromosomal aberrations were found in five patients among *ETV6-RUNX1* fusion positive cases (31.2%).

Two patients (12.5%) showed deletion of the nontranslocated *ETV6* allele. This deletion has been reported in several studies but its incidence is highly variable, ranging from 8.6% to 87.5% [2, 7, 9, 21–23].

This low detection of del(12) in our study could be explained by the fact that the *ETV6* FISH probe covered almost the entire of the *ETV6* gene so that submicroscopic deletion could be missed. 

According to the Knudson' hypothesis [9], *ETV6* could act as a tumor suppressor gene, with t(12;21) disrupting one copy of *ETV6* and the deletion being the second inactivating event [17, 21].

In the three other patients, we have found extra copies of the *RUNX1* gene in which the clone with the extra signal of RUNX1 was distinct from the one harboring *ETV6-RUNX1* fusion gene. 

Interestingly, extra copies of *RUNX1* gene were also observed in 7 patients without *ETV6-RUNX1* fusion. Of them, three do not have +21 on their karyotype which is likely consistent with extra 21 perhaps in a small clone not observed cytogenetically or derive from non dividing clonal hyperdiploid cell, while normal metaphases derived from normal dividing cells. 

In the four remaining cases, the number of *RUNX1* signals was in keeping with results on CC including a case (case 5) in which we have identified a t(3;12) by FISH. This translocation has previously been reported in a case with B-lineage ALL but in association with t(12;21) by Yehuda-Gafni et al. [17] and Kobayashi et al. [19].

Extracopies of the *RUNX1* gene were found in 6 patients (having or not *ETV6-RUNX1* fusion gene and without +21 in the karyotype) among 57 patients with B-lineage ALL (10.5%). This incidence was higher than that previously reported by Dal Cin et al. [24], Niini et al. [25] and more recently by Busson Le-Coniat et al. [26] who reported only 2 or 3 cases.

Although the real significance of these findings has not been clarified yet, the hypothesis of the gene-dosage effect involving *RUNX1* seems to be very probable [27, 28].

In summary, our findings demonstrate that *ETV6-RUNX1* fusion gene is a common genetic abnormality detected by FISH in 28% of Tunisian children with B-lineage ALL, and we confirm that secondary genetic events are commonly encountered in these patients. Interestingly, extra copies of *RUNX1* are frequently found in our series than those previously reported in other populations and so warrant further investigation to elucidate the mechanisms underlying the role of the *RUNX1* in leukemogenesis. Moreover, due to the unknown prognostic significance of this abnormality, further studies should be conducted in consecutive children with ALL to correlate *RUNX1* overexpression with the patients' followup.

## Figures and Tables

**Table 1:** Summary of patients with abnormal FISH findings.

Patients	Age/sex (years)	Karyotype (number of cells)	FISH signals (No.)			% of cells
			*ETV6*	*RUNX1*	*ETV6-RUNX1 *fusion	
1	13/M	46, XY[20]	0	1	2	15
2	2/M	46, XY[16]	0	1	1	16
3	5/M	46, XY, del(20)(p12)[8]/46, XY[10]	1	1	2	17
4	6/M	46, XY[19]	1	2	1	25
5	3/F	46, X, −X, add(3)(q27), +mar[5]/46, XX[15]	3	3	0	18
6	15/F	48, XX, t(2;11)(p12;q23), +20, +21[18]	2	3	0	24
7	13/M	46, XY, −16, +mar[9]/46, XX[6]	2	3/4	0	14
8	2/F	46, XX[20]	2	4	0	19
9	7/M	46, XY[20]	1	1	1	14
10	4/F	46, XX[21]	1	1	1	23
11	14/M	46, XY[20]	1	1	1	32
12	4/F	46, XX[21]	1	1	1	17
13	4/F	46, XX[17]	1	1	1	15
14	5/M	46, XY[21]	1	2	1	44
15	3.5/F	46, XX[19]	1	1	1	22
16	14/M	46, XX, −1, dic(1;16)(p31;p13)X2, −21, +2mar[16]	2	3	0	14
17	13/M	47, XXY, del(12)(p12 − p13)[8]/47, XXY[12]	2	3	0	16
18	7/F	47, XX, +5[9]/46, XX[3]	1	1	1	17
19	8/M	46, XY[18]	1	1	1	27
20	4/F	No metaphases	1	1	1	41
21^a^	4/F	46, XX[17]	1	1	1	18
22	3/M	46, XY[19]	1	1	1	22
23	1.2/M	46, XY[19]	2	3	0	14

^a^This case harbored two distinct clones: one with only the *ETV6/RUNX1* rearrangement and the second (9% of cells) with an extra signal of *RUNX1* gene.

No: number.
